# Global richness patterns of alpine genus *Gentiana* depend on multiple factors

**DOI:** 10.1002/ece3.11366

**Published:** 2024-05-22

**Authors:** Thae Hnin Wai, Xin Liang, Huanhuan Xie, Lian Liu, Yingji Pan, Ying Xu, Lina Zhao, Xiaoting Xu

**Affiliations:** ^1^ Key Laboratory of bio‐Resource and eco‐Environment of Ministry of Education, College of Life Sciences Sichuan University Chengdu China; ^2^ Key Laboratory of Wetland Ecology and Environment, Northeast Institute of Geography and Agroecology Chinese Academy of Sciences Changchun China; ^3^ State Key Laboratory of Black Soils Conservation and Utilization, Northeast Institute of Geography and Agroecology Chinese Academy of Sciences Changchun China; ^4^ State Key Laboratory of Systematic and Evolutionary Botany, Institute of Botany Chinese Academy of Sciences Beijing China; ^5^ China National Botanical Garden Beijing China

**Keywords:** alpine plant, environmental factor, *Gentiana*, range size, species richness

## Abstract

Environmental factors impact species richness differently across taxonomic groups, and understanding the geographic patterns and drivers influencing alpine plant richness remains limited. This study compiled global distribution data of 404 species of *Gentiana*, an alpine genus, and analyzed the relative effects of different environmental factors and several previously proposed models on the variation of *Gentiana* richness. By evaluating the effects of range size and regions on the relationships between *Gentiana* richness and environmental factors, we found that all tested environmental factors had weak effects on richness variation for all species and wide‐ranging species, while habitat heterogeneity was the best predictor for narrow‐ranging species. Habitat heterogeneity was the main driver of richness variation in Europe and Asia, but not in North America. The multiple regression model that included variables for energy, water, seasonality, habitat heterogeneity and past climate change had the highest explanatory power, but it still explained less than 50% of the variation in species richness for all *Gentiana* species at both global and regional scale, except for Europe. The limited explanatory power of environmental factors in explaining species richness patterns for all species, along with the variations observed among regions, suggest that other factors, such as evolutionary processes and biogeographic history may have also influenced the geographic patterns of *Gentiana* species richness. In conclusion, our results indicate a limited influence of climate factors on alpine species richness, while habitat heterogeneity, along with its impacts on speciation and dispersal, likely play significant roles in shaping the richness of alpine *Gentiana* species.

## Graphical abstract

Gentiana show a high species richness in Mountain regions of northern hemisphere, especially in Qinghai‐Tibet Plateau 2. Habitat heterogeneity, environmental water and past climate change have larger effects on narrow‐ranging species than wide‐ranging species 3. Multiple regression models can only explain less than 50% of the variation in species richness

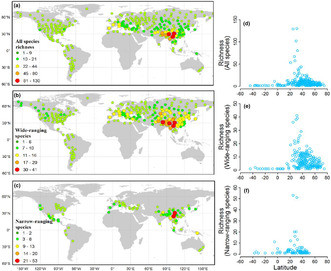

## 1 INTRODUCTION

Since the time of Darwin, ecologists and biogeographers have been interested in the observed large‐scale patterns of species richness and the underlying mechanisms (Huston, 1994). Understanding these patterns is crucial for conserving biodiversity in face of global change (Brown, 1999; Kreft & Jetz, 2007; Pennisi, 2005). Based on the relationships between species richness and environmental variables, many hypotheses, such as the ambient energy hypothesis (Allen et al., 2007), habitat heterogeneity (Stein et al., 2014), past climate change (Araújo et al., 2008), Janzen's hypothesis (Janzen, 1967), and water‐energy dynamics (Francis & Currie, 2003; O'Brien et al., 2000), have been proposed. However, the relationships between species richness and environmental variables, and the power of the above hypotheses vary among taxonomic groups, geographical regions and across range sizes (Jetz & Rahbek, 2002; Wiens & Donoghue, 2004).

The variation of richness and environment relationship among taxonomic groups may depend on the evolutionary history of target taxonomic groups (Wiens & Donoghue, 2004; Xu et al., 2019). For example, species richness of taxonomic groups originated in tropics are more likely to be limited by environmental energy, as these taxonomic groups are likely to retain their ancestral niches and lack cold adaptive abilities (Wang et al., 2012). For temperate‐originated groups, species richness can have a U‐shape curve along the environmental energy since few species could evolve the adaptive ability to neither hot nor cold climate (Xu et al., 2019). As to alpine lineages, they are likely originated or diversified in global cooling periods or during mountain uplift (Favre et al., 2016; Xing & Ree, 2017), thus may have their richness patterns negatively correlated with environmental energy due to a lack of hot adaptive ability (Liu et al., 2021; Shrestha, Su, et al., 2018). These studies evidenced the variation of species richness and climate relationship according to their evolutionary history and challenged the generality of the previously proposed hypotheses, addressing the importance of exploring the effects of environment on richness variation for a specific taxonomic group (Donoghue, 2008).

The geological history across different regions plays a significant role in influencing the extinction, speciation, dispersal, and overall shaping of the unique relationship between regional richness and environmental factors (Ricklefs, 2006). The uplift of the Himalaya‐Hengduan Mountains in eastern Asia, for instance, has been instrumental in promoting speciation and rapid radiation, resulting in a greater diversity of species (Xing & Ree, 2017). Furthermore, the intricate topography and landforms of this region have also served as refuge areas for species during past periods of climate change (Lu et al., 2018). Conversely, Europe and North America experienced greater impacts from the last glacial maximum, leading to the contraction of species' ranges and even extinction in some cases (Prentice et al., 1991; Quinzin et al., 2017). This emphasizes the necessity for more comprehensive and detailed studies on plant diversity across a wide range of environmental conditions and regional disparities.

The relationships between species richness and environmental variables vary depending on the size of their distributional ranges (Jetz & Rahbek, 2002; Lennon et al., 2004). Compared to species with narrower ranges, these species with larger range sizes tend to have better dispersal capabilities, enabling them to reach equilibrium with the contemporary climate more easily (Ebersbach et al., 2017; Jetz & Rahbek, 2002; Liu et al., 2021). These species often possess a broader niche breadth and higher genetic diversity, which had enhanced their survival during the past climate change (Sandel et al., 2011). In contrast, species with narrow ranges are typically confined to areas of high habitat heterogeneity (Jetz & Rahbek, 2002; Liu et al., 2021). They are very sensitive to climate change due to their limited dispersal capabilities, small population sizes, and low genetic diversity (Stein et al., 2014, 2015). Therefore, the richness patterns of wide‐ranging species can be more influenced by contemporary climate while narrow‐ranging species are more influenced by habitat heterogeneity and past climate change (Liu et al., 2019; Ruggiero & Kitzberger, 2004; Svenning & Skov, 2007; Tello & Stevens, 2010). However, conflicting results have been reported for alpine genus *Saxifraga*, subalpine genus *Rhododendron* and low‐land family Gesneriaceae (Liu et al., 2017, 2021; Shrestha, Su, et al., 2018). This suggests that the effects of range size on the relationships between species richness and environmental factors remain as a topic of debate.

*Gentiana* is a large genus containing over 400 species in Gentianaceae family. It grows in diverse habitats from cold Arctic tundra to warm Australia and the Andes (Ho & Liu, 2001). The Qinghai‐Tibet Plateau (QTP) is its center of diversity. Phylogenetic studies showed that the first *Gentiana* species lived on the QTP about 34 million years ago (Ma). This coincided with global climatic changes and QTP uplift (Chen et al., 2021; Favre et al., 2016). Subsequently, *Gentiana* species diversified mainly on the QTP and dispersed to all continents except for Africa. It is now prevailing in alpine meadows and withstanding extreme cold, being an idea taxon to examine the previous proposed hypotheses on alpine species richness. Here, we have assembled a comprehensive global distribution dataset comprising 404 *Gentiana* species to assess how the relationships between species richness and the environmental factors vary across different range sizes and regions for this alpine plant group.

## 2 MATERIALS AND METHODS

### 2.1 Species distribution

The global distribution of *Gentiana* species was compiled through available resources, such as checklists, monographs of field investigations for plants, peer‐reviewed articles, published flora at continental or regional scale and online databases (Appendix S1). The nomenclature of the *Gentiana* species followed Ho and Liu (2001) and was complemented by the Catalog of Life: Higher Plants in China (https://www.catalogueoflife.org/annual‐checklist/2019/). Cultivated and island species were removed. The subspecies and varieties were merged to the species level (Appendix S2). We recorded species distribution based on the geographical unit used in previous study for recording plant species distribution (Liu et al., 2021; Shrestha, Wang, et al., 2018). Our final dataset included 2439 records for 404 of *Gentiana* species distributed in 252 geographical units. The number of species in each geographical unit was counted as a proxy for species richness.

The range size was defined as the number of geographical units occupied by each species. Following previous study, we ranked all species by range size in descending order and categorized the upper 25% as wide‐ranging species and lower 50% as narrow‐ranging species (Araújo et al., 2008; Jetz & Rahbek, 2002). To test the sensitivity of the relationships between species richness and environmental factors across range size, we also categorized the upper 50% as wide‐ranging species, the lower 25% as narrow‐ranging species. However, the lower 25% range size threshold produced a very low number of distribution records (232 of 2439), which may induce high uncertainly in the following statistical analysis. Therefore, we did not perform statistical analysis for this range size group.

### 2.2 Environmental data

In this research, 22 environmental factors were tested and divided into five categories: environmental energy, environmental water, climate seasonality, habitat heterogeneity and past climate change (Table 1). Species richness is not significantly affected by area (*p* = .492). Therefore, we have excluded it in the following analysis. Contemporary climate data were obtained from the CHELSA database ver.1.2 (http://chelsa‐climate.org/) and CGIAR Consortium for Spatial Information ver.2.1 (CGIAR‐CSI, httpp://www.cgiar‐csi.org/) at a spatial resolution of 30 arc‐second (ca. 1 km at the equator). The elevation layer was downloaded from WorldClim database ver.1.4 (http://www.worldclim.org/) at a spatial resolution of 2.5 arc‐minutes. The climate of the Last Glacial Maximum (LGM) was also obtained from CHELSA database ver. 1.2 at the spatial resolution of 30 arc‐second. Soil layer data were obtained from global gridded soil information (version published in 2017, https://files.isric.org/soilgrids/former/2017‐03‐10/aggregated/1km/; Hengl et al., 2014, 2017).

**TABLE 1:** Climatic variables and their abbreviations used in the analyses.

Groups	Environmental variables	Abbreviation	Data sources (resolution)
Energy	Mean annual temperature (°C)	MAT	CHELSA ver 1.2 [30 s]
	Mean temperature of warmest quarter (°C)	MTWQ	
	Mean temperature of coldest quarter (°C)	MTCQ	
	Potential evapotranspiration (mm)	PET	CGIAR [30 s], Access time: 2017
	Minimum monthly potential evapotranspiration (mm)	PETmin	
Water	Mean annual precipitation (mm)	MAP	CHELSA ver 1.2 [30 s]
	Precipitation of driest quarter (mm)	PDQ	
	Precipitation of warmest quarter (mm)	PWQ	
	Annual actual evapotranspiration (mm)	AET	CGIAR [30 s]
	Water deficit (mm)	WD	PET‐AET
	Sum of monthly precipitation values for which mean monthly temperature was above 0 (°C)	Rainfall	Calculated
Seasonality	Temperature seasonality (°C)	TSN	CHELSA ver 1.2 [30 s]
	Temperature annual range (°C)	ATR	
	Precipitation seasonality(mm)	PSN	
Habitat heterogeneity	Elevation range (m)	ELER	Calculated using elevation from WorldClim ver1.4 [2.5 arc min]
	Range of mean annual temperature (°C)	MATR	Calculated using elevation climate from CHELSA ver 1.2 [30 s]
	Range of mean annual precipitation (mm)	MAPR	
	Soil coarse fragments volume (%)	CRFVOL	SoilGrids [1 km]
	The number of soil types within each geographical unit	NST	
Past climate change	Mean annual temperature absolute anomaly (°C)	AnomMAT	CHELSA ver 1.2 [30 s]
	Mean annual precipitation absolute anomaly (mm)	AnomMAP	

The values of each environmental variable within each geographical unit are the average of all grid cells within that unit. Range values of elevation, temperature and precipitation for a geographical unit were estimated as the difference between the maximum and minimum values of a given unit, respectively, to represent habitat heterogeneity. Past climate change was calculated as the absolute differences between LGM and present (i.e., Climate_LGM_ − Climate_present_) following Araújo et al. (2008). The number of soil types within each geographical unit was counted to represent soil habitat heterogeneity. All these calculations were performed using the zonal statistics tool in ArcGIS 10.4.1 (ESRI, Redlands, CA).

In the analysis of the relationship between species richness and environment in different regions, it was observed that South America, Australia, and Africa had species counts of less than 10, rendering them unsuitable for subsequent analysis. Therefore, here we report the results for the North American, European and Asian regions.

### 2.3 Data analysis

The skewness and the Pearson's correlation were calculated to compare the richness patterns of all *Gentiana* species, as well as wide‐ranging and narrow‐ranging species. Univariate ordinary least square (OLS) models were performed to assess the influence of each environmental factor on the richness patterns of groups with different range size and in different regions. Modified *t*‐test was used to account for the effects of spatial autocorrelation on the significance test in OLS models (Dutilleul et al., 1993). We performed the skewness, OLS and modified *t*‐test using the R packages ‘moments’, ‘stats’ and ‘SpatialPack’, respectively.

To address the issue of multicollinearity among the environmental variables, we employed the hierarchical partitioning (HP) method to assess the relative importance of each environmental category on species richness (Mac Nally, 2000). Firstly, we selected one representative variable from each environmental category. This variable exhibited a significant effect on species richness after accounting for spatial autocorrelation and demonstrated the highest explanatory power. Subsequently, HP constructed a multiple regression model using these five selected variables. The explained variance of species richness was then partitioned into the independent effects of each variable and the shared effects with other variables, effectively addressing the multicollinearity problem. The HP analysis was conducted in the R package ‘hier.part’ (Mac Nally, 2000).

Three hypotheses were tested in our study. The first hypothesis examined water‐energy dynamic using models (a) Richness ~ Rainfall + (PETmin – PETmin^2^) + log (ELER) built by O'Brien et al. (2000) and (b) Richness ~ WD + PET + PET^2^ proposed by Francis and Currie (2003). The second hypothesis, based on Janzen's hypothesis (Janzen, 1967), utilized the model (c) Richness ~ TSN + ELER. The third hypothesis involed a multiple regression model comparising Richness ~ Energy + Water + Seasonality + Habitat Heterogeneity + Past Climate Change, modified from Wang et al. (2011). In the this model, we incorporated the effects of past climate change following Liu et al. (2021). To select the final model, we considered the lowest Akaike information criterion (AIC) and highest adjusted‐*R* ^2^ values. adjusted‐*R* ^2^. These models were built using the OLS method, following the methodology outlined in the respective published articles. Prior to applying the models, species richness was log10‐transformed.

To assess the independent and shared effects of the above‐mentioned four models such as O'Brien et al. (2000), Francis and Currie (2003), Janzen (1967), and Wang et al. (2011) on richness patterns, variance partitioning analysis was carried out using the function of “varpart” in R packages ‘vegan’ (Peres‐Neto et al., 2006). All other statistical analyses were carried out in R version 3.5.1 (http://www.r‐project.org, R Core Team, 2018). Richness was log10 transformed in all OLS models. OLS, HP and VP analysis were conducted for species in each range size category and for each region.

## 3 RESULTS

**FIGURE 1:**
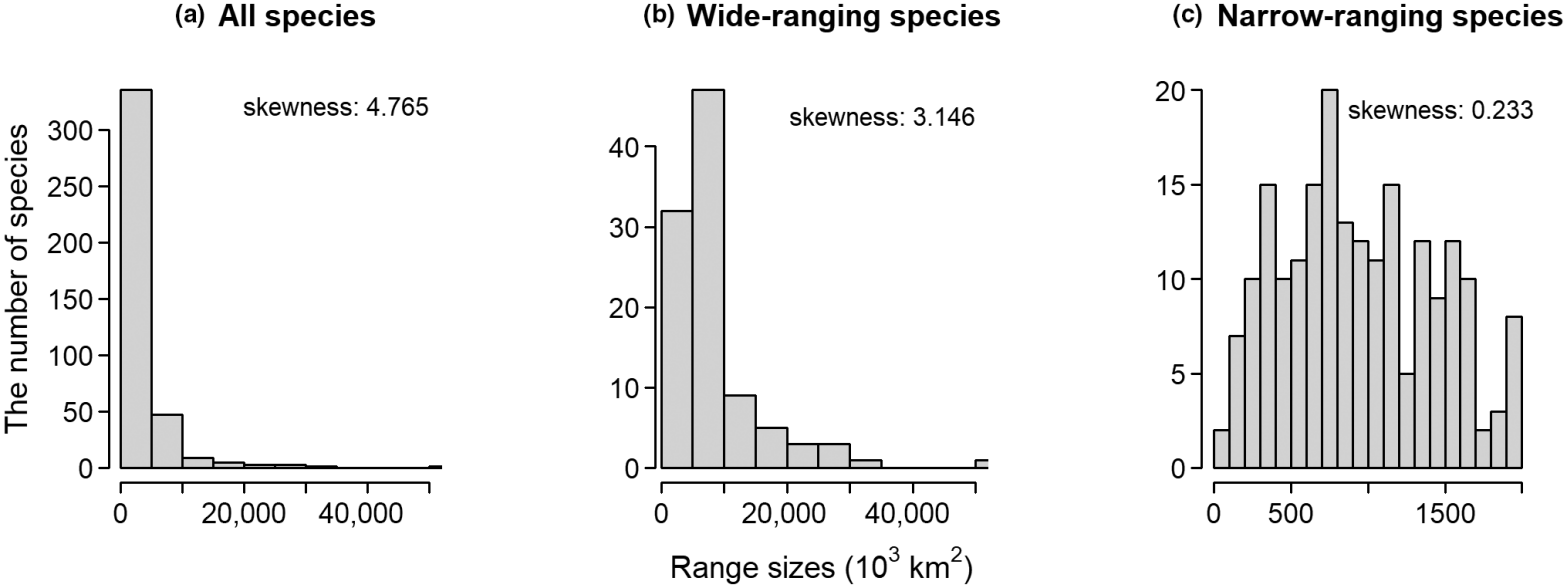
Frequency distribution of range sizes (the first line) of (a) all species, (b) wide‐ranging species, (c) narrow‐ranging species.

**FIGURE 2:**
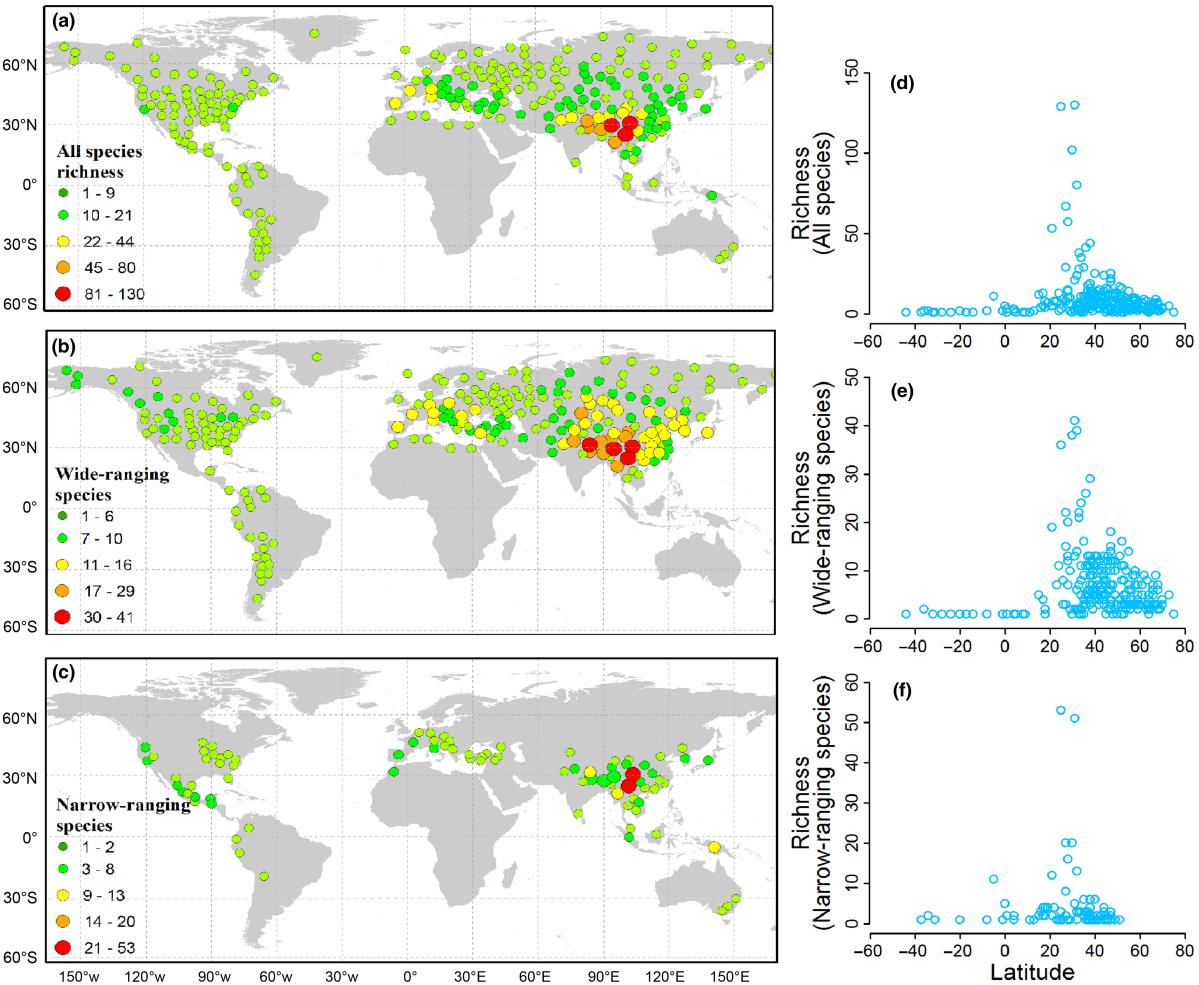
Global patterns of *Gentiana* species richness for all species (a), wide‐ranging species (b) and narrow‐ranging species (c) and the scatter plot of species richness against latitude (d, e, f).

Species range size exhibits a clear right‐skew with a skewness of 4.765 for all species (Figure 1a), 3.146 for wide‐ranging species (Figure 1b). In contrast, range size of narrow‐ranging approximates a normal distribution, with a skewness value of 0.233 (Figure 1c). Specifically, wide‐ranging species account for 63.48% of the total distribution records (i.e., 1545 of 2434 records), while narrow‐ranging species (lower 50%) represent a meer 16.2% of the total records (394 of 2434 records). *Gentiana* species predominantly inhabit the northern hemisphere, with the greatest species diversity observed in Himalaya regions around 30° N (Figure 2a,d). Wide‐ranging species are distributed continuously across tropical to temperate regions spanning from 40° S to 80° N (Figure 2b,e). Narrow‐ranging species are mainly found in mountainous regions, such as QTP, southern Europe and western North America (Figure 2c,f). The richness patterns of all species are more similar to those of wide‐ranging species (*r* = .89) than to those of narrow‐ranging species (*r* = .61). The number of species per geographical unit varies from 1 to 130 for all species, 1 to 41 for wide‐ranging species and 1 to 53 for narrow‐ranging species (Figure 2).

Our results, based on univariate OLS models, find that habitat heterogeneity is the most important factor for all species (CRFVOL: adjusted‐*R* ^2^ = 12.72%, modified *t*‐test *p* < .05, Table 2). For wide‐ranging species, environmental energy availability has the strongest negative effect (PETmin: adjusted‐*R* ^2^ = 11.24%, modified *t*‐test *p* = .022), followed by habitat heterogeneity (CRFVOL: adjusted‐*R* ^2^ = 10.33%, modified *t*‐test *p* < .1). For narrow‐ranging species, habitat heterogeneity is the main factor (ELER: adjusted‐*R* ^2^ = 27.01%, modified *t*‐test *p* = .003), followed by environmental water availability (PWQ: adjusted‐*R* ^2^ = 11.21%, modified *t*‐test *p* = .028), and past climate change (AnomMAP: adjusted‐*R* ^2^ = 7.39%, modified *t*‐test *p* = .012; Table 2). Regarding different regions, we find that habitat heterogeneity is the only significant predictor in Europe (ELER: adjusted‐*R* ^2^ = 60.78%, modified *t*‐test *p* = .020) and Asia (CRFVOL: adjusted‐*R* ^2^ = 33.49%, modified *t*‐test *p* < .01, Table 4). None of the factors are significantly correlated with species richness in North America.

**TABLE 2:** The coefficients of the standard coefficient and *adjusted‐R* ^2^ of the relationship between richness and each environmental factor for all species, wide‐ranging species and narrow‐ranging species of *Gentiana* L. based on ordinary least square models.

Groups	Predictors	All species		Wide‐ranging species		Narrow‐ranging species	
		Adjusted‐*R* ^2^ (%)	Coefficients (Coef.)	Adjusted‐*R* ^2^ (%)	Coefficients (Coef.)	Adjusted‐*R* ^2^ (%)	Coefficients (Coef.)
Energy	MAT	.50	−.042^ns^	3.47	−.079^ns^	−.56	−.033^ns^
	MTWQ	.51	−.042^ns^	1.82	−.06^ns^	4.77	−.1^ns^
	MTCQ	.61	−.044^ns^	4.23	−.086^ns^	−1.20	.003^ns^
	PET	1.68	−.064^ns^	6.37	−.104^ns^	−.91	−.022^ns^
	PETmin	3.10	−.083^ns^	11.24	−.136*	−.35	.038^ns^
Water	MAP	−.40	.003^ns^	.92	−.046^ns^	1.97	.073^ns^
	PDQ	.94	−.051^ns^	4.33	−.087^ns^	−.45	−.036^ns^
	PWQ	3.77	.09^ns^	.79	.044^ns^	11.21	.144*
	AET	−.38	.006^ns^	.66	−.042^ns^	−.51	.034^ns^
	Rainfall	−.40	−.002^ns^	1.23	−.051^ns^	.59	.055^ns^
	WD	2.62	−.077^ns^	3.17	−.075^ns^	.69	−.056^ns^
Seasonality	TSN	.14	.032^ns^	3.19	.076^ns^	1.44	−.067^ns^
	ATR	.95	.051^ns^	5.20	.094^ns^	1.35	−.066^ns^
	PSN	4.22	.095^ns^	4.01	.084^ns^	8.05	.125^ns^
Habitat heterogeneity	ELER	5.981	.11^ns^	3.63	.08^ns^	27.01	.218**
	MATR	6.38	.115^ns^	3.12	.075^ns^	21.44	.195**
	MAPR	−.20	.02^ns^	.02	−.027^ns^	8.77	.129*
	CRFVOL	12.72	.16*	10.33	.131^ns^	23.83	.205*
	NST	−.25	.017^ns^	−.41	−.006^ns^	1.57	.068^ns^
Past climate change	AnomMAT	.63	−.045^ns^	.02	−.027^ns^	8.96	−.131^ns^
	AnomMAP	.58	−.044^ns^	1.84	−.06^ns^	7.39	.12*

*Note*: *p*‐values were corrected using modified *t*‐test. Please see Table 1 for definitions of predictor abbreviations. Significance level of *p*‐value from linear regression: ***, <.001; **, <.01; *, <.05; ns, not significant.

**FIGURE 3:**
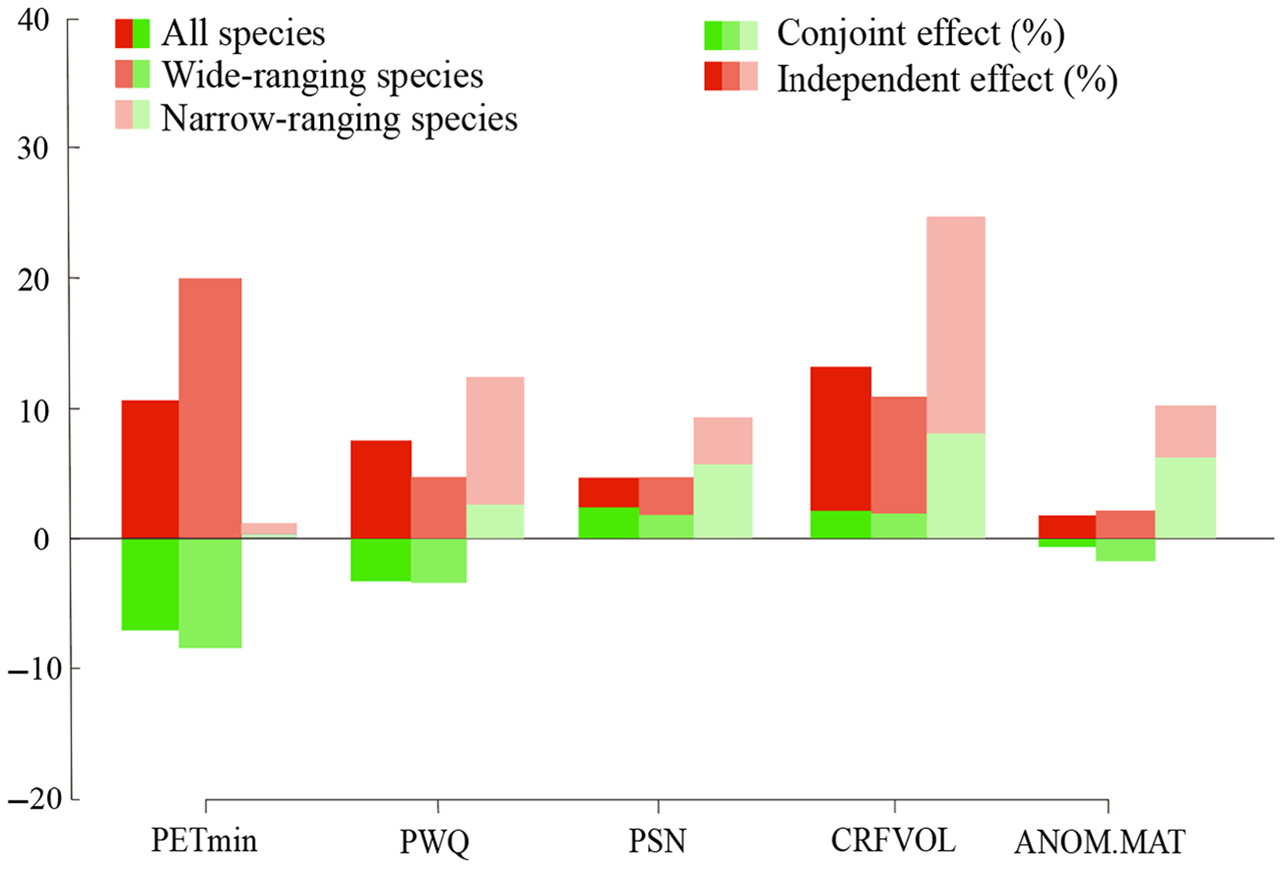
Comparison of the effects of five environmental groups on spatial variation of richness patterns for all, wide‐ranging species and narrow‐ranging species assessed by hierarchical partitioning analysis. Environmental energy (PETmin, Minimum monthly potential evapotranspiration), environmental water (PWQ, Precipitation of warmest quarter), climate seasonality (PSN, precipitation seasonality), habitat heterogeneity (CRFVOL, Soil coarse fragments volume) and past climate change (Anom.MAT).

**FIGURE 4:**
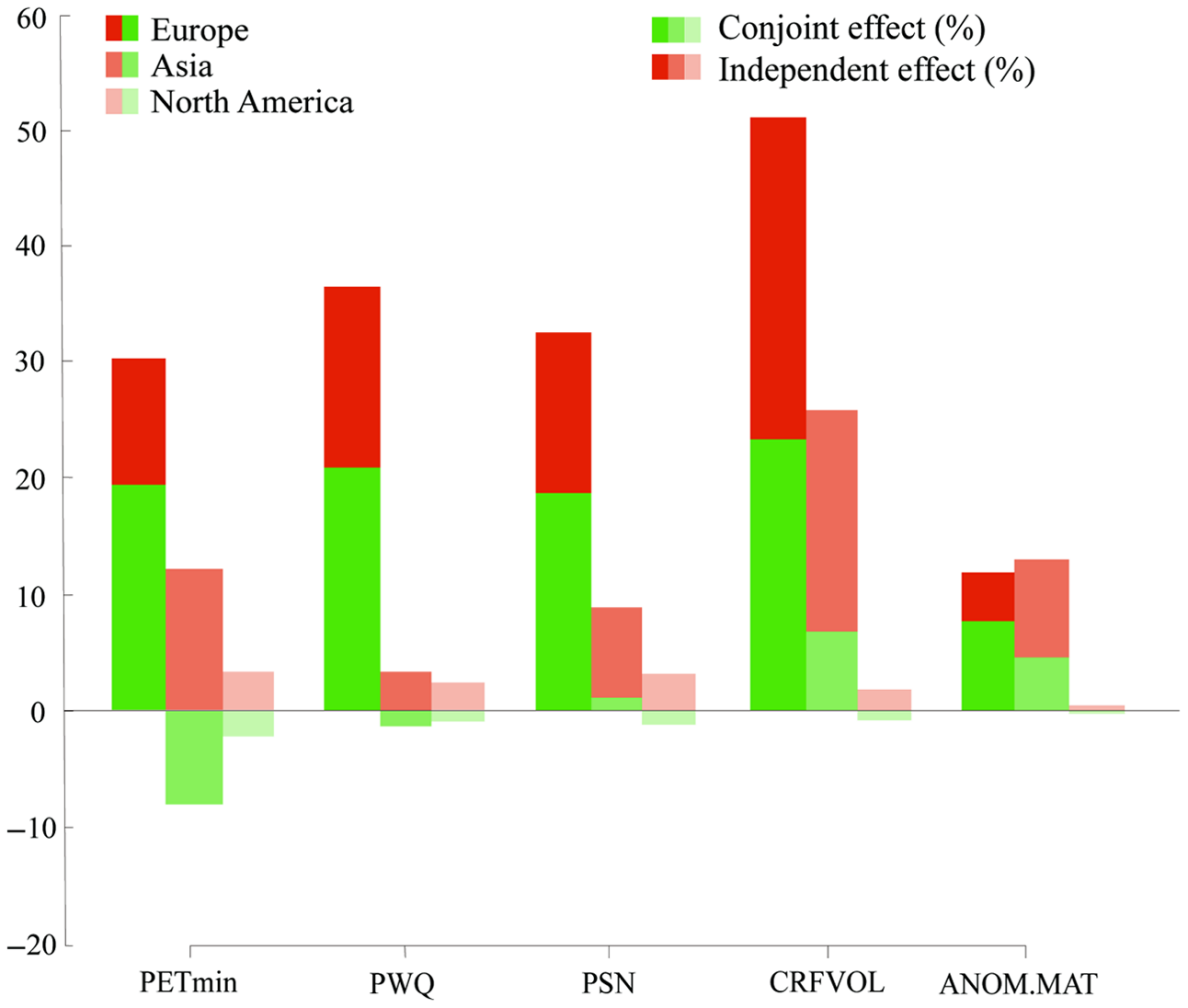
Comparison of the effects of five environmental groups on spatial variation of richness patterns for Europe, Asia and Northern America species assessed by hierarchical partitioning analysis. The five environmental groups are: Environmental energy (PETmin, Minimum monthly potential evapotranspiration), environmental water (PWQ, Precipitation of warmest quarter), climate seasonality (PSN, precipitation seasonality), habitat heterogeneity (CRFVOL, Soil coarse fragments volume) and past climate change (AnomMAT).

The HP analyses confirmed the results of the univariate models. For wide‐ranging species, environmental energy (PETmin) is the factor that had the most significant independent effects on species richness variation, while for narrow‐ranging species, habitat heterogeneity (CRFVOL) is the factor that had the most significant independent effects (Figure 3). The combined effects of all five variables are less than 10%. In the regional analyses, habitat heterogeneity (ELER) is the key variable for explaining species richness variation in Europe and Asia, with more than 20% independent effects (Figure 3). The conjoint effects of environmental energy (PETmin), environmental water (AET), climate seasonality (TSN) and habitat heterogeneity (ELER) with other factors are all more than 20% in Europe (Figure 4), resulting in a high explanatory power for species richness in the univariate OLS analysis (Table 3).

**TABLE 3:** The coefficients of the standard coefficient and adjusted‐*R* ^2^ of the relationship between richness and each environmental factor for Europe, Asia and Northern America species of *Gentiana* L. based on ordinary least square models.

Groups	Predictors	Europe		Asia		North America	
		Adjusted‐*R* ^2^ (%)	Coefficients (Coef.)	Adjusted‐*R* ^2^ (%)	Coefficients (Coef.)	Adjusted‐*R* ^2^ (%)	Coefficients (Coef.)
Energy	MAT	19.54	.185^ns^	−.33	.033^ns^	.58	.033^Na^
	MTWQ	−1.83	.031^ns^	1.41	−.067^ns^	.63	.034^Na^
	MTCQ	35.08	.242^ns^	1.4	.067^ns^	.53	.033^ns^
	PET	10.05	.14^ns^	−.58	.025^ns^	−.98	.018^Na^
	PETmin	37.69	.25^ns^	−.88	.004^ns^	−1.6	−.004^ns^
Water	MAP	32.22	.233^ns^	.13	.045^ns^	−1.12	.016^ns^
	PDQ	22.15	.196^ns^	.29	−.048^ns^	−1.55	−.007^ns^
	PWQ	1	.073^ns^	7.39	.127^ns^	−1.61	.004^ns^
	AET	44.9	.272^ns^	.27	.048^ns^	−.51	.024^ns^
	Rainfall	40.11	.258^ns^	−.29	.034^ns^	−.94	.019^ns^
	WD	−2.42	−.006^ns^	−.69	−.020^ns^	−1.62	.003^ns^
Seasonality	TSN	39.73	−.257^ns^	8.48	−.135^ns^	−.34	−.026^ns^
	ATR	29.15	−.222^ns^	4.95	−.107^ns^	−.94	−.019^ns^
	PSN	−1.97	.027^ns^	10.14	.147^ns^	−1.64	.000^ns^
Habitat heterogeneity	ELER	60.78	.314*	28.13	.238**	−.87	.020^ns^
	MATR	53.29	.295*	21.22	.208**	−.28	.026^ns^
	MAPR	40.16	.258^ns^	5.81	.114^ns^	−.32	.026^ns^
	CRFVOL	27.05	.215^ns^	33.49	.259**	−1.62	−.003^ns^
	NST	56.84	.304*	16.87	.186***	−.39	.025^ns^
Past climate change	AnomMAT	13.29	−.157^ns^	14.26	−.171^ns^	−1.62	−.003^ns^
	AnomMAP	33.69	−.237^ns^	−.2	.037^ns^	2.89	−.048^ns^

*Note*: *p*‐values were corrected using *modified t*‐test. Please see Table 1 for definitions of predictor abbreviations. Significance level of *p*‐value from linear regression: ***, <.001; **, <.01; *, <.05; ns, not significant.

Abbreviation: Na, not available.

The results of the multiple regression model reveal that the combined model of Wang et al. (2011) is the best for explaining richness variation for species with different ranges and in different regions (Tables 4 and 5). It explains approximately 40% of richness variation for all species (adjusted‐*R* ^2^ = 41.9%), wide‐ranging species (adjusted‐*R* ^2^ = 45.4%) and narrow‐ranging species (adjusted‐*R* ^2^ = 37.1%). In Europe and Asia, this model explains 73.3% and 51.7% of richness variation, respectively (Table 5). However, only 17.5% of richness variation in North America can be explained by this model. All models give high explanatory power of richness variation in Europe (adjusted‐*R* ^2^ > 50%).

**TABLE 4:** The coefficients of the standard coefficient, a*djusted‐R* ^2^ of four previous proposed models for all species, wide‐ranging species and narrow‐ranging species of *Gentiana*.

Model	Predictors	All species		Wide‐ranging species		Narrow‐ranging species	
		Coefficient	Adjusted *R* ^2^ (%)	Coefficient	Adjusted *R* ^2^ (%)	Coefficient	Adjusted *R* ^2^ (%)
O'Brien et al. (2000)	Rainfall	.238***	26.9	. 220***	33.3	.108*	30.1
	PETmin	−.050^ns^		−.152^ns^		.170^ns^	
	PETmin^2^	−.290***		−.269***		−.221^ns^	
	ELER	.187***		.168***		.228***	
Francis and Currie (2003)	WD	−.160*	16.5	−.038^ns^	25.4	−.705^ns^	−1.5
	PET	1.895***		1.819***		−.165^ns^	
	PET^2^	−1.977***		−2.152***		.151^ns^	
Janzen (1967)	TSN	.085**	8.6	.129***	10.2	−.017^ns^	26.2
	ELER	.142***		.114***		.217***	
Wang et al. (2011)	Energy	−.432***	41.9	−.395***	45.4	−.171*	37.1
	Water	.343***		.250***		.178**	
	Seasonality	.128***		.117***		−.054^ns^	
	Habitat heterogeneity	.191***		.143***		.276***	
	Past Climate Change	−.083**		−.088***		.08^ns^	

*Note*: Significance level of *p*‐value: ***, <.001; **, <.01; *, <.05; ns, not significant. Please see Table 1 for definitions of abbreviations.

Wang et al. (2011) combined model specific: all species richness ~ energy (PETmin) + water (AET) + seasonality (PSN) + habitat heterogeneity (CRFVOL) + Past Climate Change (AnomMAT); Wide‐ranged species richness ~ energy (PETmin) + water (AET) + seasonality (PSN) + habitat heterogeneity (CRFVOL) + Past Climate Change (AnomMAT); Narrow‐ranged species richness ~ energy (PETmin) + water (AET) + seasonality (ART) + habitat heterogeneity (CRFVOL) + Past Climate Change (AnomMAP).

**TABLE 5:** The standard coefficient, a*djusted‐R* ^2^ of four previous proposed models for Europe, Asia and Northern America species of *Gentiana*.

Model	Predictors	Europe		Asia		North America	
		Coefficient	Adjusted *R* ^2^ (%)	Coefficient	Adjusted *R* ^2^ (%)	Coefficient	Adjusted *R* ^2^ (%)
O'Brien et al. (2000)	Rainfall	.170^ns^	65.7	.180**	39.1	.120^ns^	0
	PETmin	1.088^ns^		.176^ns^		−.089^ns^	
	PETmin^2^	−3.790^ns^		−.395***		.023^ns^	
	ELER	.371***		.230***		.073^ns^	
Francis and Currie (2003)	WD	−1.241***	56.6	−.056^ns^	17.0	.006^ns^	10.9
	PET	−1.111^ns^		.995***		.404**	
	PET^2^	3.686***		−.980***		−.408**	
Janzen (1967)	TSN	−.218*	65.3	−.063^ns^	29.5	−.030^ns^	0
	ELER	.394***		.222***		.016^ns^	
Wang et al. (2011)	Energy	−.258***	73.3	−. 233***	51.7	.231**	17.5
	Water	.084^ns^		−. 219***		−.160**	
	Seasonality	−.192**		. 125***		.125*	
	Habitat heterogenity	.228***		.192***		.155***	
	Past Climate Change	−.182**		−.281***		−.070*	

*Note*: Significance level of *p*‐value: ***, <.001; **, <.01; *, <.05; ns, not significant. Please see Table 1 for definitions of abbreviations.

Wang et al. (2011) combined model specific: Europe species richness ~ energy (MAT) + water (AET) + seasonality (TSN) + habitat heterogeneity (NST) + Past Climate Change (AnomMAT); Asia species richness ~ energy (PETmin) + water (WD) + seasonality (PSN) + habitat heterogeneity (NST) + Past Climate Change (AnomMAT); North America species richness ~ energy (MAT) + water (WD) + seasonality (ATR) + habitat heterogeneity (MATR) + Past Climate Change (AnomMAP).

**FIGURE 5:**
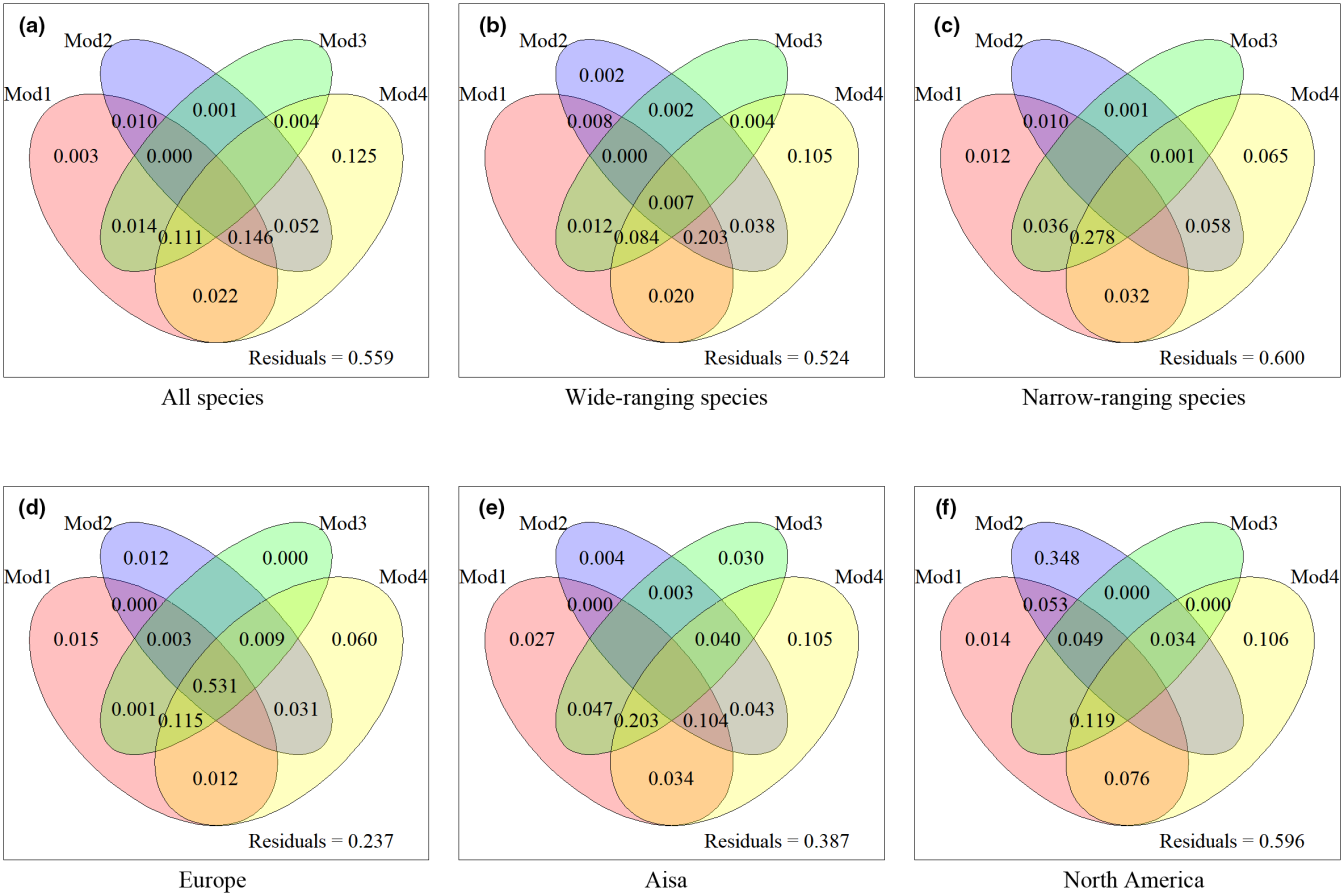
The independent and shared effects of different models on species richness variation for different groups or regions: All species (a), wide‐ranging species (b), narrow‐ranging species (c), Europe (d), Asia (e) and North America (f). Mod1 in red and Mod2 in purple represent two forms of water energy dynamic (Francis & Currie, 2003; O'Brien et al., 2000); Mod3 in green represents Janzen's hypothesis (Janzen, 1967), and Mod4 in yellow represents combined model (Wang et al., 2012) (Tables 4 and 5 for details). Values <0 not shown.

The variance partitioning analyses also reveal that the model proposed by Wang et al. (2011) independently explained the greatest variation of richness for all species (Figure 5a), species at different range categories (Figure 5b,c), and in different regions (Figure 5d–f). The shared effects of all four tested models are over 50% in Europe but less than 1% in all other groups (Figure 5). The models containing environmental energy and water have the largest shared effects for all species and wide‐ranging species (Figure 5a,b), while the models containing habitat heterogeneity show relatively large shared effects on narrow‐ranging species (Figure 5c), and species in Europe and Asia (Figure 5d,e). These results are also consistent with the univariate OLS models.

## 4 DISCUSSION

### 4.1 Habitat heterogeneity affects Gentiana species richness

Habitat heterogeneity is widely recognized as a primary factor determining species richness because of its effects on promoting speciation, providing various niches for species coexistence and serving as refugium during the last glacial maximum (Stein et al., 2014, 2015). Previous studies on alpine groups, such as the subalpine shrubby genus *Rhododendron* (Shrestha, Su, et al., 2018), the herbaceous genus *Saxifraga* (Liu et al., 2021), and *Saussurea* (Zhang et al., 2021), found that the habitat heterogeneity was either the best predictor or equally good as climate variables in explaining species richness variation. Consistent with these studies, our results indicate that the exceptionally high species richness of *Gentiana* observed in QTP and Alps regions is probably due to the high habitat heterogeneity therein. Habitat heterogeneity effects on speciation, dispersal barriers and refugium probably all contribute to high species richness in QTP and Alps regions. To begin with, biogeographical and evolutionary studies on *Gentiana* suggest that the ancestors of extant *Gentiana* species lived in the QTP and subsequently diversified therein through the allopatric speciation process associated with the uplift of Himalaya‐Hengduan Mountains (Favre et al., 2016). The diverse climate conditions that arose with the mountain uplift also provided the opportunities for *Gentiana* species to adapt to various climate, thereby facilitating the ecological speciation process. Furthermore, dispersal barriers in mountain regions with high habitat heterogeneity can also contribute to species accumulation in QTP and Alps. Only species characterized by winged seeds and capsules, such as species from the section Chondrophyllae, have managed to extend their range southward to Australia (Adams & Williams, 1988; Nathan, 2006). This limited distribution can lead to the disequilibrium in the distribution of *Gentiana* species with respect to contemporary climate, resulting in the low independent effects of present climate on global and regional *Gentiana* species richness variation. Moreover, the primary predictor of species richness variation in Europe, accounting for over 60% of the variation, is the range of elevation. This finding aligns with previous study on European trees and is likely due to the refuge provided by mountainous regions during the last glacial maximum. These regions preserved many relic species and limited their postglacial dispersal (Svenning & Skov, 2007).

### 4.2 The best model for Gentiana species richness

Of the four multiple regression models we tested here, the one proposed by Wang et al. (2011) explained the most variation in richness for all species, wide‐ranging species, and narrow‐ranging species. This model incorporated the environmental effects of energy, water, seasonality, habitat heterogeneity, and past climate change on richness patterns, unlike other models that only considered one or two factors representing a specific hypothesis. Our results suggested that no single hypothesis could adequately explain the contemporary richness patterns of alpine plant groups. These patterns may result from complex processes involving various environmental factors. However, this model could only explain about 42% of the species richness variation, which was similar to another alpine genus *Saxifagra* (Liu et al., 2021) and *Rhododendron* (Xia et al., 2022), but much lower than non‐alpine clades, especially woody plants, such as Moraceae in China (Wang et al., 2022) and *Quercus* at a global scale (Xu et al., 2019), which had over 70% of the richness variation explained. The underlying mechanisms of richness patterns of alpine groups may be much more complicated than those of lowland plants.

### 4.3 Range size effects on species richness ~ environment relationship of Gentiana

In our study on *Gentiana*, we found that contemporary climatic variables did not significantly influence the richness variation of both wide‐ranging and narrow‐ranging species after accounting for spatial autocorrelation, except for the precipitation of the wettest quarter (PWQ). PWQ explained less than 1% of the richness variation of wide‐ranging species, but 11.21% of the richness variation of narrow‐ranging species. This result contrasted with previous studies on woody plants and lowland regions (Wang et al., 2022), but was similar to *Saxifraga* (Liu et al., 2021). A recent physiological study also found that populations of the herbaceous plant *Arabidopsis thaliana* in Tibet suffered from higher drought stress compared to lowland populations in Sichuan, China (Lou et al., 2022). The narrow‐ranging species of *Gentiana*, which are mainly distributed in alpine mountain regions, might also suffer from higher drought stress than wide‐ranging species, thus showing a positive correlation with PWQ. These results suggested that there might be a distinct range size effect on the richness‐environmental water relationship of alpine groups. The richness of narrow‐ranging species of alpine groups might be more sensitive to future drying under future climate change compared to the wide‐ranging species.

Climate seasonality and past climate change since the last glacial maximum had very low explanatory power (<10%) for the richness variation of both narrow‐ranging and wide‐ranging species. A study on Saxifraga also found an insignificant effect of climate seasonality on the richness patterns of wide‐ranging and narrow‐ranging species, which was consistent with our results. However, long‐term climate change explained over 20% of the richness variation of narrow‐ranging species of Saxifraga. The low explanatory power of long‐term climate change on narrow‐ranging species of Gentiana might be because most narrow‐ranging species occurred at high elevations (>3000 m) in mountain areas and probably radiated in cold environments. Therefore, they could better adapt to and even diversify with climate cooling.

Habitat heterogeneity, as mentioned above, is a key factor affecting diversity patterns, especially for species with narrow ranges. This has been supported by many studies on a wide range of animal and plant groups (Jetz & Rahbek, 2002; Liu et al., 2019; Ricklefs & Lovette, 1999; Van Rensburg et al., 2002). Our findings are in line with these studies, demonstrating a greater impact of habitat heterogeneity on narrow‐ranging species compared to wide‐ranging species. However, in contrast to previous studies that found large effects of contemporary climate than habitat heterogeneity on wide‐ranging species, our study found that habitat heterogeneity had the highest explanatory power for wide‐ranging species of Gentiana. These results suggested that even for wide‐ranging species, their distributions are not in equilibrium with the contemporary climate. Habitat heterogeneity may play an important role in the diversity patterns of both wide‐ and narrow‐ranging species due to its effects as a barrier to dispersal and a driver of diversification.

## 5 CONCLUSION

Our results also suggested that alpine groups, which radiated with the uplift of mountains and adapted well to cold environments, might have different responses to climate and climate change than lowland groups. Habitat heterogeneity is the most important predictors of the species richness patterns of *Gentiana*. The environmental factors we investigated had very low explanatory power for the species richness variation of all and wide‐ranging species, suggesting that the distribution of *Gentiana* species may not get equilibrium with contemporary climate. Other potential factors, such as biogeographical and evolutionary history, might play an important role in shaping the contemporary richness patterns of *Gentiana* and should be explored in future studies. In conclusion, our study emphasizes the key roles of habitat heterogeneity in shaping diversity patterns of alpine plants and underscores the need for further research into the underlying mechanisms.

## AUTHOR CONTRIBUTIONS

**Thae Hnin Wai:** Data curation (equal); formal analysis (equal); writing – original draft (lead). **Xin Liang:** Writing – review and editing (equal). **Huanhuan Xie:** Formal analysis (supporting); writing – review and editing (equal). **Lian Liu:** Data curation (equal). **Yingji Pan:** Writing – review and editing (equal). **Ying Xu:** Data curation (equal). **Lina Zhao:** Data curation (equal). **Xiaoting Xu:** Data curation (equal); formal analysis (equal); funding acquisition (equal); supervision (equal); writing – review and editing (equal).

## CONFLICT OF INTEREST STATEMENT

The authors declare that they have no known competing financial interests or personal relationships that could have appeared to influence the work reported in this paper.

## Supporting information


Appendix S1.

Appendix S2.

## Data Availability

All code and data necessary to reproduce the analyses presented in this study are available at: https://datadryad.org/stash/share/KIv_TzBcHr9nFwQy6CP85futZtjjbUql0Qp7uWnZfC4.
